# Wavelength-Dependent Degradation of Ochratoxin and Citrinin by Light *in Vitro* and *in Vivo* and Its Implications on *Penicillium*

**DOI:** 10.3390/toxins4121535

**Published:** 2012-12-14

**Authors:** Markus Schmidt-Heydt, Benedikt Cramer, Irina Graf, Sandra Lerch, Hans-Ulrich Humpf, Rolf Geisen

**Affiliations:** 1 Max Rubner-Institut, Haid-und-Neu-Strasee 9, Karlsruhe 76131, Germany; E-Mails: markus.schmidt-heydt@mri.bund.de (M.S.-H.); irina.graf@mri.bund.de (I.G.); sandra.lerch@mri.bund.de (S.L.); 2 Westfälische Wilhelms-Universität, Corrensstraee 45, Münster 48149, Germany; E-Mails: cramerb@uni-muenster.de (B.C.); humpf@uni-muenster.de (H.-U.H.)

**Keywords:** ochratoxin A, ochratoxin B, citrinin, degradation, light, stability

## Abstract

It has previously been shown that the biosynthesis of the mycotoxins ochratoxin A and B and of citrinin by *Penicillium* is regulated by light. However, not only the biosynthesis of these mycotoxins, but also the molecules themselves are strongly affected by light of certain wavelengths. The white light and blue light of 470 and 455 nm are especially able to degrade ochratoxin A, ochratoxin B and citrinin after exposure for a certain time. After the same treatment of the secondary metabolites with red (627 nm), yellow (590 nm) or green (530 nm) light or in the dark, almost no degradation occurred during that time indicating the blue light as the responsible part of the spectrum. The two derivatives of ochratoxin (A and B) are degraded to certain definitive degradation products which were characterized by HPLC-FLD-FTMS. The degradation products of ochratoxin A and B did no longer contain phenylalanine however were still chlorinated in the case of ochratoxin A. Citrinin is completely degraded by blue light. A fluorescent band was no longer visible after detection by TLC suggesting a higher sensitivity and apparently greater absorbance of energy by citrinin. The fact that especially blue light degrades the three secondary metabolites is apparently attributed to the absorption spectra of the metabolites which all have an optimum in the short wave length range. The absorption range of citrinin is, in particular, broader and includes the wave length of blue light. In wheat, which was contaminated with an ochratoxin A producing culture of *Penicillium verrucosum* and treated with blue light after a pre-incubation by the fungus, the concentration of the preformed ochratoxin A reduced by roughly 50% compared to the control and differed by > 90% compared to the sample incubated further in the dark. This indicates that the light degrading effect is also exerted *in vivo*, e.g., on food surfaces. The biological consequences of the light instability of the toxins are discussed.

## 1. Introduction

Ochratoxin A is a toxic secondary metabolite produced by certain fungal species. Ochratoxin A is a composite mycotoxin with dihydroisocoumarin as the polyketide moiety coupled via peptide linkage to the amino acid phenylalanine. Ochratoxin A is chlorinated at position 5 within the dihydroisocoumarin moiety [1]. In contrast ochratoxin B has the same structure except that it does not contain chlorine but hydrogen at that position instead [1]. Ochratoxin B is by a factor of 10–100 less toxic than ochratoxin A [2] indicating that the presence of the chlorine determines toxicity. Like ochratoxin citrinin belongs to the benzopyrancarbonic acids and has a polyketide structure. The structure is very similar to the polyketide part of ochratoxin, but citrinin does not contain chlorine [3]. Both mycotoxins are mainly nephrotoxic and ochratoxin A is rated by the WHO/FAO as a type II carcinogen [4]. Ochratoxin A and citrinin may occur in the same food commodity [5] and may act synergistically [6] under a toxicological point of view.

Ochratoxin A is produced by various fungi like certain Aspergilli and Penicillia. From the Aspergilli *A. carbonarius*, *A. westerdijkiae*, *A. steynii* and *A. ochraceus* are the most important producing species [7,8]. From the genus *Penicillium* only *P. nordicum* and *P. verrucosum* are described to produce ochratoxin A [9,10]. In addition, *P. verrucosum* is also able to produce citrinin beside ochratoxin. In contrast, *P. expansum* only produces citrinin but no ochratoxin. *P. verrucosum* is the ultimate species responsible for the occurrence of ochratoxin and citrinin in wheat [11]. According to these authors a contamination of wheat by *P. verrucosum* to a certain extend unequivocally indicates the presence of ochratoxin A.

Nevertheless the amount to which an individual species produces the toxins is dependent on the environmental conditions. It was previously shown that light as an environmental factor has a profound influence on mycotoxin biosynthesis. Light, especially blue light, reduces the biosynthesis of ochratoxin in *P. nordicum* and *P. verrucosum* [12] and in *Aspergillus niger* [13] but increases citrinin biosynthesis in *P. expansum* and *P. verrucosum* [12] ochratoxin B production in *P. nordicum* [14] and fumonisin biosynthesis in *A. niger* [13] and *F. proliferatum* [15]. 

To get further insight into the role of the adaptive changes of the secondary metabolite profiles under light conditions, the stability and the absorption profiles of the three mycotoxins ochratoxin A, ochratoxin B and citrinin were determined under various light wavelengths. It was shown that short wavelength light has a profound effect on the stability of all three secondary metabolites, however citrinin was affected most. Ochratoxin was degraded to definitive products which were characterized by high performance liquid chromatography coupled with fluorescence detection and fourier transform mass spectrometry (HPLC-FLD-FTMS). Moreover, it was shown that short wavelength light can reduce and control the amount of preformed ochratoxin A in wheat. These findings are discussed in view of the possible adaptive biological and ecological effect of the produced mycotoxins under light conditions.

## 2. Results

### 2.1. Influence of Light on the Stability of Various Mycotoxins

**Figure 1:**
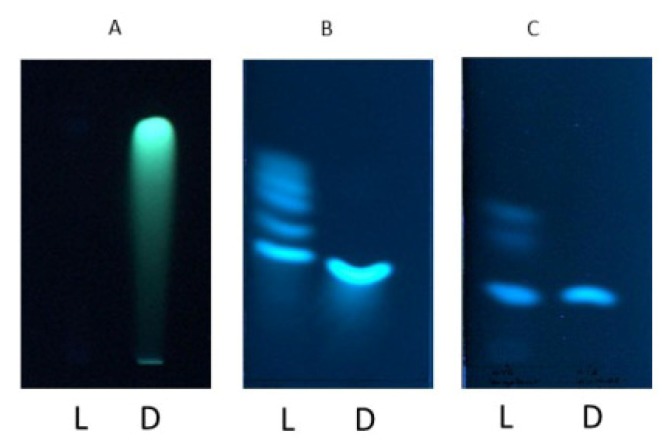
Thin layer chromatography of citrinin (**A**); ochratoxin A (**B**) and ochratoxin B (**C**) of the respective mycotoxins in methanol (10 µg/mL ochratoxin A (OTA)/ochratoxin B (OTB), 200 µg/mL citrinin) after incubation for 24 h at 25 °C in the dark (**D**) or under white light (L) of an intensity of 366 µW/cm^2^.

To analyze if light has an influence on the stability of the secondary metabolites ochratoxin A/B or citrinin, methanolic solutions of these toxins were placed into white light (366 µW/cm^2^) and incubated for 24 h. After that time the samples were analyzed by TLC and the spots visualized by UV light (Figure 1). Interestingly, all treated mycotoxins showed signs of light dependent degradation. 

The least degradation was found with ochratoxin B, because it shows the highest amount of the non-degraded original metabolite ochratoxin B. Citrinin in contrast was nearly completely degraded, because no residual toxin could be detected on the TLC plate. However, it cannot be ruled out that citrinin is degraded to non-detectable metabolites (non-fluorescent) under the TLC conditions used. Compared to ochratoxin B a clearly smaller amount of ochratoxin A withstand the 24 h light treatment indicating a reduced stability of ochratoxin A compared to ochratoxin B. 

In contrast to citrinin, ochratoxin A and ochratoxin B were degraded to defined degradation products because new fluorescent bands occurred after light treatment. The chlorine at position 5 in the dihydroisocoumarin moiety [1] of ochratoxin apparently has an important impact on the light dependent stability because a different set of fluorescent bands appeared after the light treatment of both toxins. For ochratoxin A four degradation bands and for ochratoxin B two degradation bands could be separated after TLC. Subsequent analysis by HPLC-FLD-FTMS using an excitation wavelength of 330 nm and an emission wavelength of 460 nm revealed one major degradation product of ochratoxin A and three products with low signal intensity. Using the same fluorescence parameters, four degradation products of ochratoxin B could be determined, each with comparable signal intensity.

The fact that after a light treatment of ochratoxin A and B for 24 h still residual amounts of toxin could be identified is most likely dependent on the current conditions, e.g., the original amount of toxin used for this study, the intensity of the light and the duration of the treatment.

### 2.2. Influence of Light Wavelength on the Degradation Capacity

**Figure 2:**
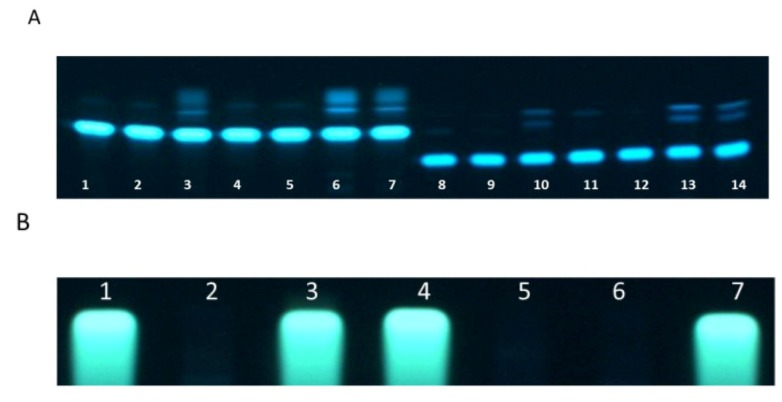
Thin layer chromatography of ochratoxin A (lanes 1–7) and ochratoxin B (lanes 8–14) incubated for 24 h at 25 °C in the dark (lanes 1 and 8), under red (lanes 2 and 9), white (lanes 3 and 10), green (lanes 4 and 11), yellow (lanes 5 and 12), royal blue (lanes 6 and 13) and blue (lanes 7 and 14) (**A**); The degradation of both mycotoxins after treatment with white, blue and royal blue light can be seen. Thin layer chromatography of citrinin (**B**) incubated for 24 h at 25 °C under red (lane 1), white (lanes 2), green (lanes 3), yellow (lanes 4), royal blue (lanes 5), blue (lanes 6) and in the dark (lane 7). The complete degradation of citrinin under white, blue and royal blue light is obvious.

In order to analyze which light wavelength is responsible for the observed effects, the same experiment was carried out except that light of particular color and thereby wavelength was used. Figure 2 shows the result of this analysis with ochratoxin A and B. 

Degradation of ochratoxin A (Figure 2A, lanes 1–7) and ochratoxin B (Figure 2A, lanes 8–14) only took place after treatment with white, blue (470 nm) and royal blue (455 nm) light. For all other tested conditions (red (627 nm), yellow (590 nm) and green (530 nm)) none, respectively much less degradation was detectable. The same results could be found after treatment of citrinin by light (Figure 2B). In this case an even complete degradation of citrinin took place after exposure to white, blue and royal blue light. 

These results clearly demonstrates, that the active parts of the white light, which leads to a degradation of the analyzed mycotoxins, are the blue parts from 470 to 455 nm. All other light components are inactive (or much less active), with respect to degradation. All inactive light components had a longer wave length than the active blue light. It can therefore be assumed that light with a further reduced wave length will have an even higher potential to degrade the toxins.

### 2.3. Characterization of the Degradation Products of Ochratoxin A and B by HPLC-FLD-FTMS

**Figure 3:**
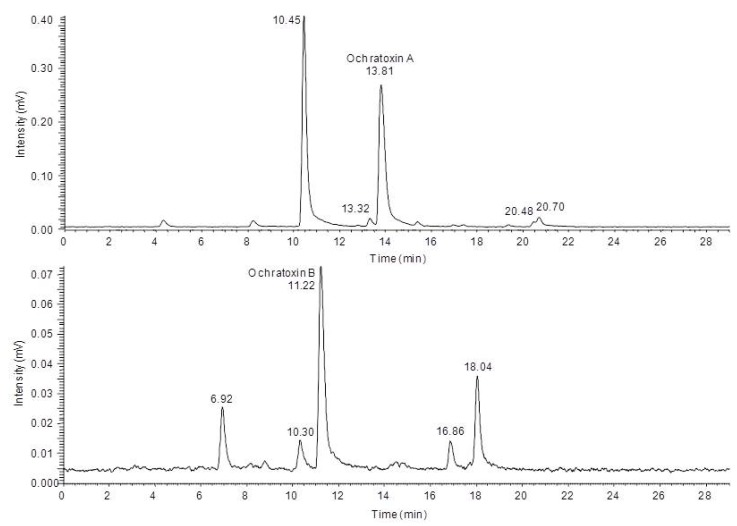
High performance liquid chromatography-fluorescence detection (HPLC-FLD) chromatograms of ochratoxin A (top) and ochratoxin B (below) after 24 h irradiation with blue light.

In order to characterize the different degradation products which occur after light treatment of either ochratoxin A or ochratoxin B a HPLC-FLD-FTMS analysis was carried out. With this system it was possible to record high resolution MS spectra and, if the signal intensity was sufficient, also to record unit resolution MS^2^ to MS^4^ spectra of the fluorescent compounds during a HPLC run. Based on these data as well as data from in source fragmentation of the mass spectrometer, a characterization of the chemical modifications of the major degradation products was possible, allowing a mass spectrometric structure assignment of the new compounds. This analysis revealed that in all cases modifications or cleavages at the phenylalanine moiety occur after blue light irradiation of ochratoxin A and B. Chromatograms of the HPLC-FLD-FTMS analysis of ochratoxin A and ochratoxin B after irradiation are shown in Figure 3. 

In Table 1 the determined exact mass signals from the chromatograms and the assigned sum formulas of the four degradation products of ochratoxin A are shown. 

**Table 1:** Light induced ochratoxin A degradation products detected by high performance liquid chromatography-fluorescence detection-fourier transform mass spectrometry (HPLC-FLD-FTMS).

Retention time [min]	Detected *m*/*z *(ionization polarity)	Calculated sum formula of the ion	Calculated *m*/*z*	Deviation [ppm]	Difference to Ochratoxin A
10.45	256.0371 (positive)	(C_11_H_10_O_4_NCl+H)^+^	256.0371	−0.1	–C_9_H_8_O_2_
13.32	282.0173 (negative)	(C_12_H_10_O_5_NCl−H)^-^	282.0175	−0.5	–C_8_H_8_O
20.48	360.0995 (positive)	(C_19_H_18_O_4_NCl+H)^+^	360.0997	−0.7	–CO_2_
20.70	358.0839 (positive)	(C_19_H_16_O_4_NCl+H)^+^	358.0846	−0.5	–CH_2_O_2_

**Figure 4:**
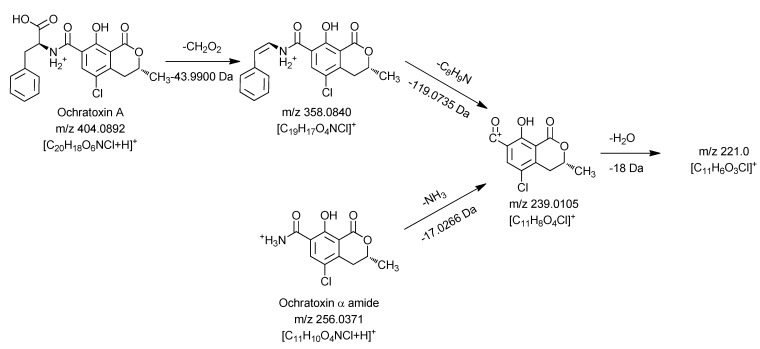
Mass spectrometric fragmentation pathway of ochratoxin A and ochratoxin α amide using positive ionization mode.

For all new compounds the characteristic chlorine pattern could be observed. The first peak at 10.45 min is the predominant signal in the chromatogram and corresponds to ochratoxin A after a loss of C_9_H_8_O_2 _Subsequent in-source and ion trap fragmentation of this compound with positive ionization revealed the fragmentation pathway shown in Figure 4. 

Similar to the MS^3 ^and MS^4^ fragments of ochratoxin A, this compound gave *m/z* 239.0105 and *m/z* 221.0 as key MS^2^ and MS^3^ signals. Thus it can be concluded that the dihydroisocoumarin structure of the degradation product is identical with the respective part in the ochratoxin A molecule (ochratoxin α). Based on these data, the amino function present in the molecule can only be connected as an amide moiety, allowing an assignment of the peak at 10.45 min to ochratoxin α amide.

**Figure 5:**
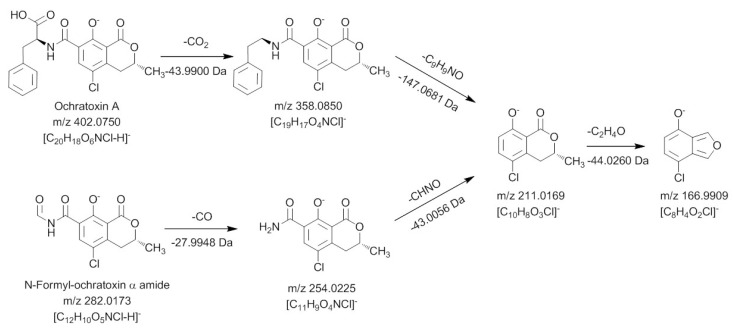
Mass spectrometric fragmentation pathway of ochratoxin A and *N*-formyl-ochratoxin α amide using positive ionization mode.

The second ochratoxin A degradation product eluting at a retention time of 13.32 min could only be sufficiently detected with negative ionization. Compared to ochratoxin α amide, an additional CO unit is attached to the molecule. Due to the strong difference in the ionizability of this compound compared to ochratoxin α amide, it could be assumed that the amino function of this molecule must be modified. Fragmentation and comparison with the signals obtained for ochratoxin A in the negative ionization mode confirmed these assumptions and are shown in Figure 5. 

In a first collision reaction, CO is cleaved from the compound leading to *m*/*z* 254.0225, which is subsequently fragmented in MS^3^ and MS^4^ experiments to *m/z* 211.0169 and *m/z* 166.9909, respectively. Both ions are also characteristic for the dihydroisocoumarin moiety of ochratoxin A, showing that the carbonyl must be connected to the amino function of the molecule. Thus, this ochratoxin A degradation product is *N*-formyl-ochratoxin α amide. The third light induced degradation product of ochratoxin A, eluting at a retention time of 20.48 min, could be assigned as 14-decarboxy-ochratoxin A, using an available reference standard [16]. Due to the low amount, it was not possible to perform sufficient fragmentation experiments of the fourth degradation product with a retention time of 20.70 min, to allow structure elucidation. However, compared to ochratoxin A, a CH_2_O_2_ group is cleaved, making the formation of a desaturated 14-decarboxy-ochratoxin A the most probable explanation.

The analysis of the light induced degradation products of ochratoxin B revealed parallels to the degradation products of ochratoxin A. However, also new modifications were detected and the concentrations of the individual compounds were very different compared to ochratoxin A. As shown in Table 2, also four degradation products for ochratoxin B could be detected and analyzed. 

**Table 2:** Light induced ochratoxin B degradation products detected by HPLC-FLD-FTMS.

Retention time [min]	Detected *m*/*z* (ionization polarity)	Calculated sum formula of the ion	Calculated *m*/*z*	Deviation (ppm)	Difference to ochratoxin B
6.92	222.0760 (positive)	(C_11_H_11_O_4_N+H)^+^	222.0761	−0.2	–C_9_H_8_O_2_
10.30	248.0565 (negative)	(C_12_H_11_O_5_N−H)^-^	248.0656	0	–C_8_H_8_O
16.86	340.1180 (positive)	(C_19_H_17_O_5_N+H)^+^	340.1179	+0.1	–CH_2_O
18.04	324.1230 (positive)	(C_19_H_17_O_4_N+H)^+^	324.1230	0	–CH_2_O_2_

The two degradation products eluting at 6.92 min and 10.30 min are the non-halogenated analogs of the first two compounds detected for ochratoxin A. In both cases, the MS^2^ to MS^4 ^fragments were identical with ochratoxin B. For the first compound, ochratoxin β amide, ions in the positive mode with *m*/*z* 205.0494 (MS^2^) and *m*/*z* 187 (MS^3^) were recorded, with the loss of NH_3_ being the first fragmentation reaction. The fragmentation of *N*-formyl-ochratoxin β amide in the negative ionization mode with a retention time of 10.30 min resulted in the loss of CO to *m*/*z* 220.0616 (MS^2^) followed by a cleavage of the amide function to *m*/*z* 177.0560 or the loss of water to *m*/*z* 202.0511 (both MS^3^). 

The structures of the two light induced degradation products of ochratoxin B with the retention times of 16.86 min and 18.04 min could not be assigned. However, based on the previous observations, it can be assumed that most likely the loss of CH_2_O and CH_2_O_2 _take place at the free carboxylic acid function.

### 2.4. Absorption Maxima of Ochratoxin A/B and Citrinin

**Figure 6:**
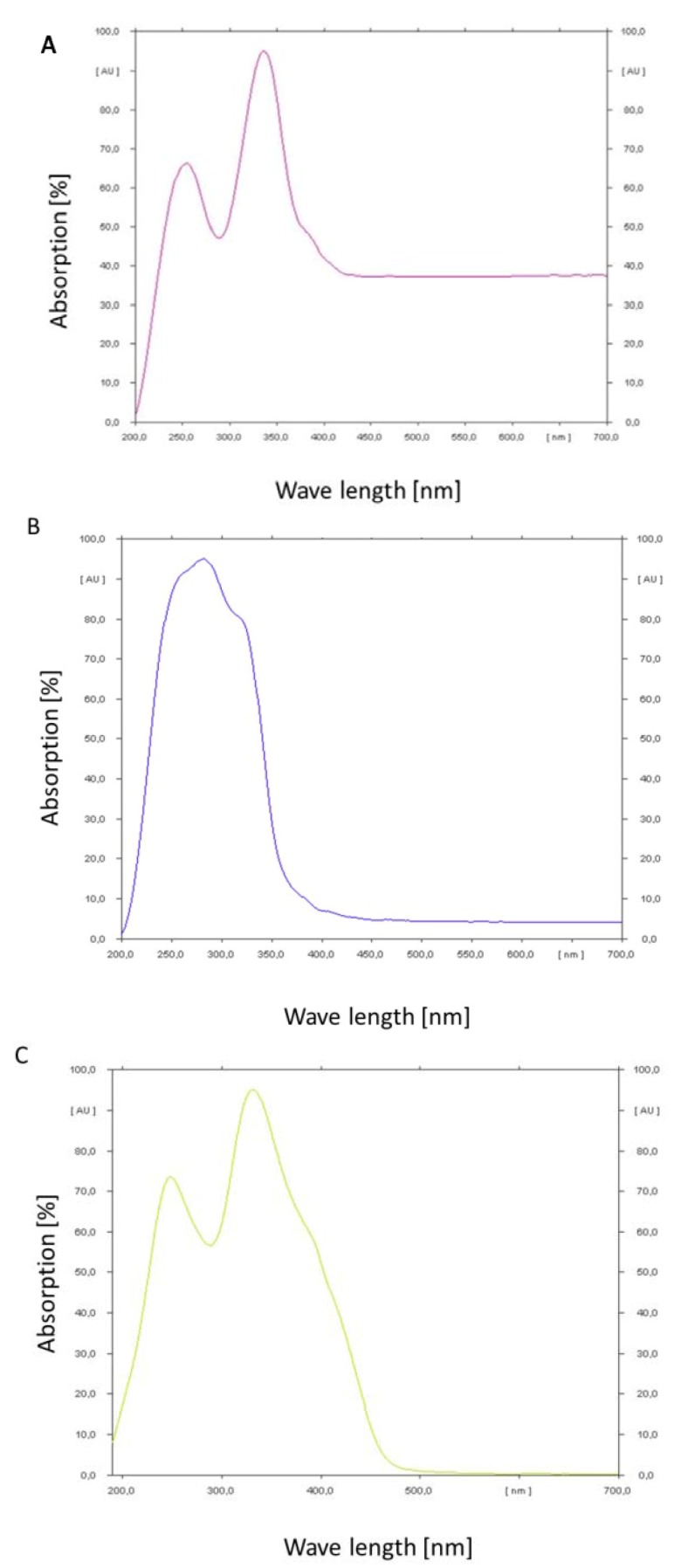
Absorption spectra determined by a CAMAG thin layer chromatographic scanner for ochratoxin A (**A**); ochratoxin B (**B**) and citrinin (**C**).

In order to determine the absorption maxima of the mycotoxins a thin layer chromatogram was performed and the absorbance of the spots was measured under changing wavelength with a spectrum analysator (TLC Scanner III, Camag, Muttenz, Switzerland). The results are shown in Figure 6A–C. 

Ochratoxin A shows a double peak of absorption in the range of 200 to 400 nm. The maximum peak is at 350 nm. At wavelengths higher than 400 nm a constant absorption of about 37% could still be observed. Ochratoxin B in contrast shows a maximum and steep peak at 270 nm. The peak covers the range from 200 to 380 nm. Above this wavelength a constant absorption of only 4% could be observed. Citrinin has a broad double peak from below 200 nm up to 480 nm. The optimum is around 330 nm. At wavelength higher than 480 nm almost no absorption could be observed. According to these results ochratoxin A and citrinin show the highest absorbance in the wavelength range of blue light, e.g., 455 to 470 nm, which is in contrast to ochratoxin B which has the least absorbance in this wave length range. That is in congruence with the relative stability of the analyzed three secondary metabolites under light irradiation.

### 2.5. Degradation of Preformed Ochratoxin A and Ochratoxin B in Wheat by Treatment with Blue Light

**Figure 7:**
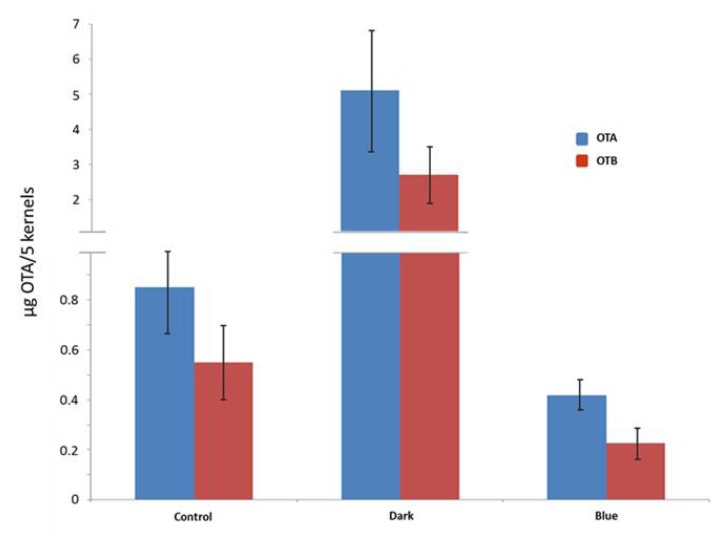
Quantitative HPLC analysis of wheat contaminated with an ochratoxin A producing *P. verrucosum* strain. The wheat was incubated for 2 weeks at 25 °C under high humidity conditions to ensure ochratoxin A biosynthesis (control). After incubation the samples were divided. One of the samples was stored in the dark for further 5 days (dark). The other was treated with blue light of 455 nm (blue). The ochratoxin A and B concentration was determined by HPLC.

In order to analyze if the observed *in vitro* degradation of ochratoxin A can also be observed under *in vivo* conditions, e.g., in a typical food environment where ochratoxin A can regularly be found, wheat were inoculated with *P. verrucosum* and the change in the concentration of ochratoxin A after incubation of the sample in the dark or under blue light conditions were determined by HPLC as described in Materials and Methods (Figure 7). 

The control sample shows the concentration of ochratoxin A after the pre-incubation of 2 weeks. In the sample incubated further in the dark a clear strong further increase of the ochratoxin A content of >80% compared to the starting concentration could be observed. This was apparently due to the unimpeded growth and metabolism of the ochratoxin A producing *P*. *verrucosum* which was physiologically active under these conditions. In sharp contrast the sample incubated under blue light did not show any increase in ochratoxin A or B but a decrease of about 50% instead. This indicates that by blue light not only are the growth and the activity of the fungus inhibited, but also preformed ochratoxin A is degraded. Ochratoxin B behaved very similar, albeit at lower concentrations.

## 3. Discussion

It has previously been shown, that the biosynthesis of ochratoxin A and citrinin by *Penicillium* is dependent on light [12]. The production of ochratoxin A is strongly reduced under light, especially blue light, whereas the biosynthesis of citrinin is increased under these conditions. In the present work we demonstrate that also the toxin molecules themselves are strongly affected by light and this has implications towards the producing organism itself. All three secondary metabolites are degraded after incubation in white light. Whereas the fluorescent citrinin signal entirely disappeared from the chromatogram indicating a complete degradation, ochratoxin A and ochratoxin B are degraded to definitive products. These degradation products obviously do no longer carry the phenylalanine in both cases. They however carry chlorine in the case of ochratoxin A but of course none in the case of ochratoxin B. In the current experiments ochratoxin was partly degraded, but it can be assumed that a complete degradation can be achieved if the light intensity or the treatment time is increased. From the spectrum of the visible light only the short wavelength range, e.g., the blue range from 470 to 455 nm were active in degrading all analyzed secondary metabolites. This is mainly in congruence with the spectra of the three metabolites itself which all has the highest absorption in the low wavelength range. The absorption spectrum of citrinin has the strongest overlap with the effective blue light wave lengths which can explain the high sensitivity of this toxin. The optimum of the absorption spectra of ochratoxin A and B however are at a lower wave length, e.g., in the UV range. A treatment with light of wavelengths below 455 nm may even further degrade these toxins. This however was not the topic of this work because UV has a profound influence on food components. Recently, Moreau *et al.* [17] analyzed the influence of pulsed white light on the degradation of various mycotoxins. They showed that zearalenone, deoxynivalenol, aflatoxin B_1_ and ochratoxin can be degraded by this treatment. They also analyzed the degradation fragments of ochratoxin A by LC-MS/MS and found two ions after the application of one flash. One correspond to the molecule after the removal of water the other after removal of formic acid. In the current analysis the HPLC-FLD-FTMS analysis revealed four different degradation products in the case of ochratoxin A which could be identified as the new compounds ochratoxin α amide, *N*-formyl ochratoxin α amide and 14-decarboxy-ochratoxin A. Furthermore, an unsaturated analog of 14-decarboxy-ochratoxin was detected. Based on the fluorescence, ochratoxin α amide is the predominantly formed compound. In the case of ochratoxin B four major degradation products were also detected. Two structures of these degradation products could be elucidated as ochratoxin β amide and *N*-formyl-ochratoxin β amide. However, the results obtained here cannot be compared directly with the results of Moreau *et al.* [17] because completely different light irradiation conditions have been applied.

This light dependent degradation of ochratoxin A and citrinin has consequences on the physiology and ecology of the producing fungus which ends up in adaptation processes by shifting the metabolite profile under light conditions. It was shown that in *P. nordicum* ochratoxin A biosynthesis is shifted towards ochratoxin B, whereas the secondary metabolite profile in *P. verrucosum* is shifted from ochratoxin A towards citrinin under light conditions [12,14].

Ochratoxin A contains a dihydroisocoumarin moiety coupled to phenylalanine. The results described here show that phenylalanine is cleaved by illumination with blue light. That results in a chloride containing coumarin derivative. It was recently shown that chlorinated coumarins have strong antifungal properties [18] and are even used as novel seed protectants [19] against fungi. This means that light produces toxic substances in cultures of ochratoxin A producing Penicillia and in fact a toxic activity of ochratoxin A on *Penicillium* under light irradiation have been demonstrated [14]. Ochratoxin B in contrast is degraded to non-chlorinated coumarin derivatives. These compounds are not toxic and even have antioxidant properties [20,21]. A shift of the production of ochratoxin A after growth in the dark to ochratoxin B after growth under light conditions therefore might have two positive functions for *P. nordicum*. Firstly, the degradation products of blue light are much less toxic and secondly light can induce oxidative stress [22], which may be counteracted by the antioxidative reactivity of non-chlorinated coumarin derivatives.

In case of *P. verrucosum* no ochratoxin B but increased amounts of citrinin are produced under light conditions. Concomitantly the ochratoxin A biosynthesis is reduced [12]. It was previously suggested that citrinin may be a light protectant [23]. *P. expansum* which only produces citrinin and no ochratoxin A drastically increases its production under blue light conditions. Interestingly, the growth of *P. expansum* as a high citrinin producing organism is only marginally affected by blue light [12] which supports the hypothesis by Stormer *et al.* [23] that citrinin may play a role as light protectant. This is further supported by the absorption spectrum of citrinin, which covers the blue light range of 470–455 nm. Irradiation of citrinin by blue light leads to a complete disappearance of the signal after TLC separation which might be a hint for a complete degradation. In this case the citrinin molecule apparently absorbs a high amount of the energy of the blue light which may result in a protective mechanism. It was further recently shown that citrinin also has antioxidative properties [24]. Light induces oxidative stress [22] and an antioxidative activity of citrinin is a further hint for the protective status of citrinin under light conditions. The rapid degradation of citrinin by light however demands an immediate replacement of the degraded citrinin by the fungus. This is apparently achieved by the induction and activation of citrinin biosynthesis under blue light. In fact, Wang *et al*. [25] described an induction of the citrinin biosynthesis genes in *Monascus* by blue light, indicating that indeed the fungus replaces the degraded citrinin by an increased production.

The degrading activity of blue light was also tested with ochratoxin A contaminated wheat. A wheat sample with an actively growing ochratoxin A producing *P. verrucosum* culture was applied to blue light irradiation. The observed results show a controlling effect of blue light. Apparently, the ochratoxin A production of *P. verrucosum* is immediately stopped after placing the wheat under blue light. Moreover, part of the preformed ochratoxin A was also degraded under these conditions. This situation indicates that the irradiation of *P. verrucosum* contaminated wheat by blue light leads to two activities: firstly the inactivation of *P. verrucosum* itself which stops further ochratoxin A biosynthesis and secondly the degradation of preformed ochratoxin A (by about 50% in the described experiment. In experiments in which only a single layer of kernels were treated with blue light to ensure a homogeneous and continuous irradiation even a higher degradation could be achieved). Presumably, the described degradation products are being formed also in the wheat system. Until now no information about their toxicity against higher eukaryotic organisms is known. Of course it has to be kept in mind that blue light only acts on the surface of the product and that the outcome of the treatment is strongly dependent on the actual conditions (light intensity, treatment time, homogeneous distribution, initial contamination). In view of the fact that citrinin is much more sensitive against blue light it can be assumed that this toxin is even more degraded in food commodities than ochratoxin.

Taken together, the results demonstrated here show that the three toxins citrinin, ochratoxin A and ochratoxin B are differentially sensitive to blue light and can be degraded to a level depending on the actual conditions. This was shown *in vitro* and *in vivo*. Together with results already described [12,14] the biological and ecological relevance of the light dependent degradation of the toxins could be elucidated. 

## 4. Experimental Section

### 4.1. Strains and Growth Conditions

*P. verrucosum* BFE808 (BFE: strain collection of the Max Rubner Institut, Karlsruhe, Germany) were used for the wheat contamination experiments. This strain is able to produce ochratoxin A and citrinin and was originally isolated from wheat. For the active production of ochratoxin A in wheat, 100 g of wheat kernels were put in a glass beaker and inoculated with 1 mL of a spore solution (10^5^/mL) of *P. verrucosum.* After inoculation the wheat was mixed thoroughly and placed in the incubation chamber at 25 °C under high humidity conditions for 20 days. To generate the spore solution *P. verrucosum* was grown on YES medium agar plates (20 g/L yeast extract, 150 g/L sucrose, 15 g/L agar) for 10 days at 25 °C in the dark. Spore suspensions were prepared by suspending spores from the YES grown colonies in TWS (0.1% Tween 80, 0.85 g/L NaCl).

For the analysis of light on the stability of preformed ochratoxin A an amount of 200 g of wheat in a glass beaker were inoculated with 2 mL of a spore solution of *P. verrucosum* BFE808 (10^6^ spores/mL) and incubated at 20 °C in the dark for 5 days in an incubation chamber. In parallel a tray of water was placed into the chamber to ensure high relative humidity in the chamber. Each day the wheat was mixed to ensure homogeneous growth of the fungus on the wheat kernels. After 5 days of this pre-incubation for the contamination of wheat with ochratoxin A, the samples were divided into two beakers with each 100 g of contaminated wheat. One sample was placed in the dark and incubated further at 20 °C for 5 days. The other sample was placed under blue light for 5 days at 20 °C. Both samples were mixed each day.

### 4.2. Treatment of Toxin Solutions with Light of Different Wavelength

From each mycotoxin the following solutions in 500 µL methanol were prepared: citrinin, 100 µg; ochratoxin A, 5 µg; ochratoxin B, 5 µg. The mycotoxins were purchased from Sigma (Deisenhofen, Germany). An amount of 500 µL was transferred in a transparent micro reaction tube and placed into a light box which contains different chambers equipped with LEDs emitting light with different wavelength, e.g., white light (366 µW/cm^2^), red (627 nm, 1127.3 µW/cm^2^), green (530 nm, 1061.4 µW/cm^2^), yellow (590 nm, 937.0 µW/cm^2^) blue (470 nm, 345.1 µW/cm^2^) and royal blue (455 nm, 490.4 µW/cm^2^). The emission characteristics of each LED, which all represents a single sharp peak, are given in Table 3. The white light was generated by a blue LED which was vaporized with phosphorus. This resulted in an emission spectrum with a sharp peak at about 440 nm (blue luminescence, minimum wavelength of this peak about 420 nm, maximum and about 475 nm) and a further long and smooth peak (optimum 550 nm) from 475 to about 780 nm (phosphorescence). 

**Table 3:** Emission conditions of the colored LEDs.

Colour	Minimum^1^ [nm]	Optimum ^1^ [nm]	Maximum ^1^ [nm]
Red	620	627	645
Yellow	585	590	597
Green	520	530	550
Blue	460	470	490
royal blue	440	455	460

^1^ the data represents the emission spectra of each colored LED. The column “Minimum” gives the wavelength where the peak begins, “Optimum” gives the wavelength with the highest emission and “Maximum” gives the maximum wavelength were the peak ends.

The exact features of the light box have been described by Schmidt-Heydt *et al*. [12]. The light box was placed in a temperature controlled room of 25 °C and the samples were illuminated for up to 5 days. As a control, a sample was incubated in the dark at the same temperature. After that time the treated samples and the control sample were analyzed by TLC or HPLC-FLD-FTMS. 

### 4.3. Thin Layer Chromatography (TLC) and HPLC Analysis

For determination of ochratoxin A, B and citrinin biosynthesis, 1 g of the crushed wheat kernels were placed into 2 mL micro reaction tubes and 1 mL of chloroform was added. The samples were extracted for 30 min at room temperature on a rotary shaker; the solid particles were discarded and the chloroform extract was evaporated to dryness in a vacuum concentrator (Speed Vac, Savant Instruments, Farmingdale, NY, USA). The residues were re-dissolved in 100 µL methanol and 20 µL were spotted onto TLC plates (Silica gel 60, Merck, Darmstadt, Germany). Alternatively 20 µL of the light treated toxin solutions were applied. As mobile phase toluol:methanol:acetic acid (90:5:5, *v*:*v*:*v*) was used for ochratoxin and toluol:ethylacetate:formic acid (60:30:10, *v*:*v*:*v*) for citrinin. Pure ochratoxin A/B or citrinin was used as standard. The spots were visualized under UV light (254 nm). In case of quantitative determination of ochratoxin A/B by HPLC, the re-dissolved residues were subjected to HPLC according to the following conditions. HPLC analysis was performed on a Shimadzu D-7000 system (Shimadzu, Duisburg, Germany) equipped with an auto-injector, column oven and fluorescence detector. The column oven was set to 25 °C, the fluorescence detector was set to an excitation of 333 nm and an emission of 460 nm. Separation was carried out on a LiChrospher 100, C_18_ (250 mm, Ø 4 mm i.d., particle size 5 µm) reversed phase column (VWR International GmbH, Darmstadt, Germany). Solvent A consisted of 48% ACN, solvent B of 1% acetic acid and solvent C of H_2_O. The flow rate was set to 1.0 mL/min and the injection volume was 20 µL. The limit of detection was 10 pg on column. Data collection and handling was done with EZ-Chrome Elite 3.2.

### 4.4. Characterization of the Ochratoxin A/B Degradation Products by LC-FLD-MS

HPLC-FLD-FTMS analysis was done on an LTQ-Orbitrap XL Fourier transform mass spectrometer coupled to an Accela LC with Accela Pump 60057-60010 and Accela Autosampler 60057-60020 (Thermo Scientific, Dreieich, Germany). An additional fluorescence detector FPl150 (Jasco, Groß-Umstadt, Germany) was coupled between the column and the heated ESI source of the mass spectrometer. Data acquisition was performed with Xcalibur version 2.0.7 SP1 (Thermo Scientific, Bremen, Germany). Chromatographic separation of the ochratoxins and the degradation products was carried out on a 150 mm × 2.1 mm i.d., 5 μm, Gemini-C_18_ column with a 4 × 2 mm guard column of the same material (Phenomenex, Aschaffenburg, Germany) using a linear binary gradient at a column temperature of 40 °C and an injection volume of 10 μL. Solvent A was methanol containing 5 mM ammonium acetate and solvent B water containing 5 mM ammonium acetate. The HPLC was programmed isocratically for the first 2 min at 20% A and a flow rate of 250 µL/min, followed by a linear gradient to 70% A in 18 min. Afterwards, the column was washed with 70% solvent A for 3 min at an increased flow rate of 350 µL/min and equilibrated for 6 min with 20% A and a linear decrease of the flow rate to 250 µL/min. 

The mass spectrometer was run under the following conditions: Capillary temperature was 225 °C, vaporizer temperature, 350 °C; sheath gas flow, 50 units; auxiliary gas, 10 units; source voltage, +3.5 kV/−3.5 kV; and tube lens, +110 V/−110 V for positive and negative ionization mode, respectively.

One scan event was programmed to perform a total ion scan of *m/z* 150–1000 at a resolution of 30.000 and a maximum injection time of 100 ms. Fragmentations of the analytes were performed after the detection of the parent ions of the peaks of interest in the high resolution scans. The detected *m/z* were noted on a parent mass list, which was used by the software to select parent ions for fragmentation in the ion trap with a maximum injection time of 250 ms, a normalized collision energy of 35 arbitrary units and an isolation width of 2.50. The most intense peak from each spectrum was also fragmented under similar conditions to achieve MS^3^ data. Repetition yielded the MS^4^ data shown below.

### 4.5. Determination of the Absorption Maxima of Ochratoxin A/B and Citrinin

To determine the absorption maxima of the different mycotoxins, 20 µL of each mycotoxin were plotted onto a SIL G-25 TLC silica plate (Macherey-Nagel, Düren, Germany) with an automated TLC Sampler III (CAMAG, Muttenz, Switzerland). The mobile phase used was toluol: ethyl acetate: formic acid (60:30:10). The toxin bands were detected under a TLC visualizer (CAMAG, Muttenz, Switzerland) and the absorption spectra were determined with a TLC Scanner III (CAMAG, Muttenz, Switzerland). The software package used for spectrum analysis was the winCATS Planar Chromatography Manager, Version 1.4.4.6337 (CAMAG, Muttenz, Switzerland).

## 5. Conclusions

The mycotoxins ochratoxin A/B and citrinin are unstable under light, especially blue light conditions. The degradation products of ochratoxin and the intermediate degradation steps were elucidated. Ochratoxin A is degraded to chlorine containing degradation products. Citrinin is apparently completely degraded. It is also shown for the first time that blue light can be used to control ochratoxin biosynthesis in wheat as the natural habitat of *P. verrucosum*. The degradation of ochratoxin A/B and of citrinin has biological implications on growth and physiology of *P. verrucosum* and apparently contributes to environmental adaptation processes.
